# To Compare the Methods of Pregnancy Termination for Fetal Abnormality in the First and Second Trimesters

**DOI:** 10.5402/2012/843245

**Published:** 2012-05-06

**Authors:** H. S. Wong

**Affiliations:** Australian Women's Ultrasound Centre, Brisbane, QLD 4109, Australia

## Abstract

Fetal abnormality is a major cause of termination of pregnancy and preservation of the fetus is important for confirmation of the diagnosis. Various regimes have been reported for termination of pregnancy for fetal abnormality in the first and the second trimesters. In this paper, we compare those regimes that allow preservation of the fetus, in terms of the efficacy in expulsion of the fetus, the factors and the side effects.

## 1. Introduction

Fetal abnormality is known to be a major cause of perinatal mortality. Termination of pregnancy for fetal abnormalities significantly decreases perinatal mortality resulting from birth defects [1, 2]. Fetal morphological anomalies (without chromosomal anomalies) and chromosomal anomalies form the main ground for termination of pregnancy in 39% and 35% of cases respectively in a study by Guillem et al. [3] and 47% and 33%, respectively, in another study by Ramalho et al. [4]. For morphological anomalies identified by ultrasound with no evidence for abnormal karyotype, it is estimated that autopsy adds information that leads to a refinement of the risk of recurrence in 27% and revision to a higher risk of 1 in 4 in 8% of cases [5]. In another study, complete correlation between ultrasound findings and pathological examination is found in only 61.1% of autopsies. This highlights the importance of pathological examination for confirmation [4]. When surgical termination is employed for fetal abnormality, an intact fetus is not obtained. Pathological examination of fetal parts by use of radiography, gross dissection, microscopic examination, and/or cell culture for karyotyping or biochemical analysis may detect a major abnormality in 92%, and in 46% a specific diagnosis was obtained only from pathologic examination [6]. As there is a trend for prenatal diagnosis to take place earlier in pregnancy, largely as a result of first trimester ultrasound screening [7], more pregnancies are expected to be terminated in the first trimester. Preservation of the whole fetus is possible with medical termination in the first trimester [8–11] although pathological examination is usually technically more difficult in these specimens [12]. In this paper, we compare the methods and outcome for termination of pregnancy for fetal abnormality in the first and second trimesters, respectively, especially those that potentially allow preservation of the fetus for pathological examination. 

## 2. Method and Materials

Medline was searched for induced abortion (MeSH) and prenatal diagnosis, and also for induced abortion (MeSH) for fetal abnormality. The first search resulted in 604 hits and the second 29 hits. The papers in English language were reviewed for any description and outcome analysis on termination of pregnancy for fetal abnormality in the first and second trimesters. Further search was performed by hand from related or cited articles.

## 3. Results

### 3.1. Termination in the Second Trimester for Fetal Abnormality

Kanhai and Keirse studied the use of low dose sulprostone in termination of pregnancy for fetal abnormality between 16 and 35 weeks in 32 pregnancies [13]. In 31 pregnancies, intravenous infusion of 0.5 mg sulprostone per minute was given for 60 minutes followed by 1 mcg per minute until the fetus was expelled. All patients delivered vaginally. 84% delivered within 36 hours. The median induction-expulsion interval was 23 hours. In one patient, 0.5 mg of endocervical prostaglandin E2 was given followed by infusion of up to 2 mcg sulprostone per minute. This patient delivered vaginally after 74 hours. Induction-expulsion intervals were not associated with gestational age. Manual removal of placenta was required in 28% of cases. Median blood loss was 100 mLs. 9% patients lost more than 1000 mLs. Six out of 32 patients (18%) did not require analgesic and 6 patients (18%) experience gastrointestinal side effects.

Hinshaw et al. studied the use of 600 mg mifepristone priming followed by misoprostol 36–48 hours later (800 mcg vaginally then 400 mcg orally 3-hourly, maximum 4 doses) in 20 cases of mid-trimester termination for fetal abnormality [14]. All patients aborted within 15 hours. The median induction-to-abortion interval was shorter for parous women compared with nulliparous women (5.5 versus 11.25 hours). Medical termination of pregnancy was complete in 95% with only one patient requiring uterine evacuation. Intramuscular opiates were administered in 12 (60%) and 2 other required nonnarcotic analgesics alone (10%). The remaining 30% did not require analgesia. Ten women (50%) developed nausea and 8 (40%) had vomiting. Four patients (20%) noted diarrhoea. There was no case of infection and 9 (45%) developed prostaglandin-associated pyrexia (<38°C).

Dickinson and Evans compared regimes incorporating oral or vaginal misoprostol in a randomized trial for termination of pregnancy at 14–26 weeks for fetal abnormality [15]. They found that the use of vaginal misoprostol 400 mcg every 6 hours (*n* = 28) was 1.9 times more likely to result in delivery within 24 hours compared with oral administration (400 mcg orally every 3 hours, *n* = 29) (rate of delivery 86% versus 45%). Moreover, no further intervention was required to effect delivery 48 hours after commencement of misoprostol in the vaginal administration group compared with 20.7% in the oral group. The former group had less vomiting score and the latter group had more diarrhoea. There was no difference in women's perception of the termination process.

Dickinson reviewed the outcome of termination of pregnancy with misoprostol at 14–28 weeks for fetal abnormality in 101 women with 1 to 3 prior Caesarean section(s) against 609 women with unscarred uterus in a cohort (median gestation 19.4 versus 19.3 weeks) [16]. Despite that the former group consisted of older women with increased parity, there was no statistical significant difference in abortion duration or complications including blood loss and blood transfusion. There was no case of uterine rupture or hysterectomy.

The addition of mifepristone for priming (200 mg 24–48 hours prior, *n* = 199) to misoprostol alone (400 mcg every 6 hours, maximum 48 hours, *n* = 189) in a sequential cohort for termination at 14–24 weeks (median 19 weeks) was found to reduce the median abortion duration from 15.5 to 8.6 hours and the duration of hospitalisation from 31.5 to 27.2 hours [17]. Increasing gestation was associated with a longer duration of termination regardless of the regime used (6.5 hours for 14–18 weeks and 10.5 hours for 20–28 weeks in the mifepristone priming group). Complications including fever, blood loss, rate of blood transfusion (2% versus 3.7%) and the rate of placental retention requiring removal in theatre (25.6% versus 24.5%) was not statistically significantly different. In both groups, about 10% of women had blood loss of ≥500 mLs. There was no case of uterine rupture or hysterectomy in this series.

The factors influencing the duration of pregnancy termination with vaginal misoprostol (400 mcg vaginally 6-hourly) for fetal abnormality at gestational age ≥13 weeks were studied in a consecutive case series involving 1066 women [18]. The median gestation at termination was 19.5 weeks and the median abortion interval was 16.1 hours. Lower maternal age (median duration 17.6 versus 15.2 versus 13.6 hours for age <30 versus 30–39 versus >40 years, *P* < 0.001), nulliparity (median duration 19 versus 14.3 hours for multiparous women, *P* < 0.001), and increasing gestation (median duration 13 versus 17.8 hours, at <16 and >20 weeks resp., *P* < 0.001) were associated with abortion prolongation. Multiparous women also required less misoprostol to effect delivery. 164 (15.4%) women had at least one prior Caesarean section (all lower segment) and 25% had more than one Caesarean scar. There was one uterine rupture in a woman with 2 previous Caesarean sections. The specific fetal anomaly did not influence the abortion duration after controlling for gestation, age, and parity, with the exception of musculoskeletal abnormalities (*P* = 0.03). Although prolonged induction was noted in termination of pregnancy with fetal neural tube defect or hydrocephalus in a previous study [19], it was not confirmed in this study. Placental retention occurred in 31.1% of cases, being 53.2% at <16 weeks gestation compared with 40.6% and 12.8% at 16–19 weeks and ≥20 weeks, respectively. Overall blood loss and the need for transfusion increased with placental retention. In 5 cases (0.47%) alternative methods were required to achieve termination of pregnancy either due to failed misoprostol induction (*n* = 4) or uterine rupture (*n* = 1).

Lo et al. retrospectively compared the effect of gestational age on the outcome of 280 second-trimester termination of pregnancies for fetal abnormalities using vaginal misoprostol 400 mcg 3-hourly, up to 5 doses in 24 hours [20]. It was noted that termination before 17 weeks was associated with higher chance of incomplete abortion (43% versus 25%, OR 2.2) and lower chance of experiencing significant side effects compared to termination after 20 weeks (2% versus 11%). All cases aborted within 72 hours, with 83.9% in the first day.

In a retrospective study involving termination of 184 singleton pregnancy for fetal abnormalities in late first and second-trimester termination of pregnancy (11–24 weeks), prostaglandin derivatives, either 200 mcg misoprostol (orally and vaginally at the same time) or 1 mg gemeprost (4–6 hourly), was used in patients without uterine scar. 1 mg gemeprost or 0.5 mg dinoprostone 4–6 hourly was used in cases of previous Caesarean section. The median induction-expulsion interval was 18 hours and the interval was longer than 24 hours in 32.1%. Gestational age and past history of spontaneous delivery were significant contributing factors to induction interval of ≤24 hours [21].

These studies are summarised in Table 1.

In one study, the effect of feticide on the duration of labour induction was examined retrospectively and there was no significant difference noticed in the outcome except that the group with feticide tend to have more procedure in the form of manual extraction of placenta or uterine curettage or both (82.9 versus 65.6%, *P* = 0.01) [22]. This study differs from the other studies above in that it is not stratified according to the method of termination but rather whether feticide was performed or not. It is therefore not included in the summary table.

There were 2 studies that stratify the outcome according to the type of fetal abnormality and they are also excluded in this review [19, 23]. 

### 3.2. Termination in the First Trimester

There is no specific study on termination of pregnancy for fetal abnormality in the first trimester although case reports have indicated success with the use of prostaglandin vaginally [8–11].

In a Cochrane systemic review, a comparison was made between medical and surgical methods for first trimester termination of pregnancy [24]. Four different interventions were compared: prostaglandin alone, mifepristone alone, and miferistone/misoprostol and methotrexate/misoprostol versus vacuum aspiration. These trials were not for fetal abnormality alone. The efficacy rates were ranging between 76% and 97.2% for medical (the lowest being prostaglandin alone group) and between 94 and 100% for surgical abortions in the individual trials. The combination of mifepristone followed by a prostaglandin is the most common used medical method for first trimester abortion. There was no statistical significant difference in the blood loss between mifepristone/misoprostol group versus vacuum aspiration although longer duration of bleeding, more vomiting and diarrhoea, more pain following the procedure and more uncompleted abortion (OR  2.1, not reaching statistic significance) was noted with the former. More patients in the surgical termination group (79%) preferred to have the same method in the future compared to the medical group (70%). However, the results were derived from small trials. There is inadequate evidence to comment on the acceptability and side effects of medical compared to surgical first-trimester abortions.

In another Cochrane review on medical methods for first trimester abortion, combined regimens are noted to be more effective than single agents [25]. Misoprostol vaginally is more effective than orally and the dosage can be lowered to 200 mg in combined regimen.

In cervical preparation for first trimester surgical abortion, mefipristone 200 mg 24 hours prior to the procedure has superior results when compared to misoprostol 600 mg given sublingually 2-3 hours prior to procedure [26]. Gemefrost had similar effect as laminara tents.

## 4. Discussion

The study by Kanhai and Kierse [13] illustrated the possible transference of methods of termination of pregnancy for other indications, for example fetal death, to fetal abnormalities. It appears that the methods used for termination of pregnancy in the first and second trimesters are also transferrable [14, 15, 17, 25–27].

The trend now is to administer prostaglandin orally or vaginally. Vaginal prostaglandin is shown to be more effective than oral. Priming with mefipristone gives better results. The effects of gemeprost and mifepristone on the mechanical properties of the cervix prior to first trimester termination of pregnancy had been studied in the past it was shown that both were similarly effective in increasing cervical distensibility. However cervical dilatation was easier with a 48 hour regimen of mifepristone than with gemeprost [27]. Therefore a combination of mifepristone and vaginal prostaglandin appears to be the most effective method.

When it comes to first trimester termination of pregnancy for fetal abnormality, there are 2 main areas of concern:

1. How certain are we of the diagnosis and the severity of affection?
2. What are the natural history and the spontaneous loss rate of the condition?

Understanding of embryonic development and transitory changes is required to avoid false positives. Moreover there are certain limitations to first trimester scans [12]. In termination of pregnancy up to 9 weeks for social reasons, pathological examination revealed that 19% had a structural abnormality and 34% of cases might have ended in a failed pregnancy [28]. In chromosomal abnormalities diagnosed at 11–14 weeks, it is estimated that 46% of cases of Trisomy 21 and 83% of cases of trisomy 18 and 13 will result in intrauterine death [12].

Another area that may be explored on is the psychological effects of termination of pregnancy. This is outside the scope of the present review.

## 5. Conclusion

Termination of pregnancy for fetal abnormality in the first and second trimesters is feasible with those methods that are used for termination of pregnancy in general and yet preserving the fetus for pathological examination. The combination of mefipristone and vaginal misoprostol appears to give the best results. Termination at an earlier gestation is associated with a shorter duration of induction but an increased chance of incomplete abortion.

## Figures and Tables

**Table 1:** Comparison of the methods for termination of pregnancy for fetal abnormality in the first and second trimester.

Study	Methods of termination of pregnancy	No. of patients	Gestational age (weeks)	Median/ mean GA (weeks)	Type of study	Patients' characteristic	Median duration (hours)	Duration > 24 h (%)	Blood loss (mLs)	Incomplete abortion (%)	Analgesia required	Side effects
Kanhai and Keirse [13]	Sulprostone IV 0.5 mg/min for 60 min then 1 mcg/min	31/32	16–35		Case series		23 hours, Not related to gestational age		Median 100 mLs, 9% > 1000 mLs	28%	82%	18% GI

Hinshaw et al. [14]	Mifepristone 600 mg po then misoprostol after 36–48 hours, 800 mcg PV then 400 mcg po q3h, max 4 doses	20			Case series		5.5 hours multiparous, 11.25 hours nulliparous^†^			5%	70%	Up to 50% GI

Dickinson and Evans [15]	Misoprostol PV 400 mcg q6h, or	28	14–26	19.6	Randomized clinical trial		14.5^†^	14.3%^†^	NP	42.9%	75%	Adjunctive methods required in 0%,
	Misoprostol po 400 mcg q3h, or	29		19.4			25.5^†^	55.2%^†^		34.5%	69%	20.7%, and
	Misoprostol 600 mcg PV then 200 mcg po q3h	27		20.5 (mean)			16.4^†^ Parity and previous CS have no effects	25.9%^†^		30.8%	77.8%	3.7% after 48 h GI side effects higher with oral misoprostol

Dickinson [16]	Misoprostol in 6 different regimes, most commonly 400 mcg q6h	720	14–28	19.4	Cohort	Previous CS (101) versus unscarred uterus (619)	16.6	17.8%	7.9% > 500 mLs	41.6%		adjunct methods required in some patients
				19.3			14.5	23.9%	5.6% > 500 mLs	34.4%		No case of uterine rupture

Dickinson et al. [17]	Misoprostol 400 mcg q6h (max 48 h) versus	189	14–24	19.6	Cohort	Multiparous in 60.3% and	15.5^†^ (16.8 nullip 14.1 multip)	21.7%	150 mLs	24.9%	66.1%	Duration of hospitalisation 31.5 h versus 27.2 h^†^
	Mifepriston 200 mg po then	199		19.1		57.8%	8.6^†^ (11 nullip 6.9 multip)	8.5%	100 mLs	25.6%	74.9%	
	Misoprostol 800 mcg PV 24–48 hours later then 400 mcg po q6 h (max 5 doses/day, 48 h)					20.9% had ≥1 previous CS	Nulliparity and advancing gestation increase induction-abortion interval^†^		About 10% ≥ 500 mLs in both groups			

							16.1 overall		100 mLs when placenta not retained	31.1% overall,		
Dickinson and Doherty [18]	Misoprostol PV 400 mcg q6h	1066	13–28	19.5	Cohort, excluding the patients in publication dated 2003	Multiparous in 60.2% 15.4% with ≥1 previous CS Stratified according to types of fetal anomaly	19 nullip 14.3 multip	23%		53.2% at <16 weeks,		Induction-abortion interval ≥48h in 3.7%, 50% of which requiring adjunct methods.
							13 at <16 weeks 17.8 at ≥20 weeks		200 mLs when placenta retained	40.6% at 16–19 weeks,		Failure of medical termination in 0.47% including 1 case of uterine rupture (1/164 or 0.6% with previous CS, 0.09% overall).
									9.4% ≥ 500 mLs	12.8% at ≥20 weeks^†^		
							Younger maternal age, nulliparity and advancing gestation increase induction-abortion interval			Median dosage to effect delivery 1200 mcg (3 doses), higher for nullip		Maternal fever ≥37.8°C

Lo et al. [20]	Misoprostol 400 mcg q3 h, max 5 doses/24 hours	280	13–23	NP	Retrospective	Stratified to <17 weeks (69),	NP (stated not statistically different between groups)	16.1%	NP	43%	62%	Significant side effects in 2%,
						17–20 weeks				31%		8%
						(121) and >20 weeks (90)				25%		11%

Wagner et al. [21]	Misoprostol 200 mcg po and PV q4-6 h, or	184	11–24	19.2	Retrospective	Induction-expulsion interval and delivery < 24 h stratified against various factors	18 overall, 15 for misoprostol	32.1% overall, increasing GA and no previous spontan-eous delivery as predictors	NP	NP	NP	NP
	Gemeprost 1 mg q4–6h, or						20 for gemeprost					
	dinoprostol 0.5 mg q4–6 h with previous CS						64 for dinoprostol					

GA: gestational age, PV: per vaginum, po: per oral, mcg: microgram, mg: milligram, ml: millilitre, h: hour, NP: not provided, ^†^: statistical significant, max: maximum, CS: Caesarean section, nullip: nulliparous, multip: multiparous, GA: gestational age.

## References

[1] Bourke J, Bower C, Blair E, Charles A, Knuiman M (2005). The effect of terminations of pregnancy for fetal abnormalities on trends in mortality to one year of age in Western Australia. *Paediatric and Perinatal Epidemiology*.

[2] Zimmer EZ, Avraham Z, Sujoy P, Goldstein I, Bronshtein M (1997). The influence of prenatal ultrasound on the prevalence of congenital anomalies at birth. *Prenatal Diagnosis*.

[3] Guillem P, Fabre B, Cans C, Robert-Gnansia E, Jouk PS (2003). Trends in elective terminations of pregnancy between 1989 and 2000 in a French county (the Isère). *Prenatal Diagnosis*.

[4] Ramalho C, Matias A, Brandão O, Montenegro N (2006). Critical evaluation of elective termination of pregnancy in a tertiary fetal medicine center during 43 months: correlation of prenatal diagnosis findings and postmortem examination. *Prenatal Diagnosis*.

[5] Boyd PA, Tondi F, Hicks NR, Chamberlain PF (2004). Autopsy after termination of pregnancy for fetal anomaly: retrospective cohort study. *British Medical Journal*.

[6] Klatt EC (1995). Pathologic examination of fetal specimens from dilation and evacuation procedures. *American Journal of Clinical Pathology*.

[7] Snijders RJM, Johnson S, Sebire NJ, Noble PL, Nicolaides KH (1996). First-trimester ultrasound screening for chromosomal defects. *Ultrasound in Obstetrics and Gynecology*.

[8] Wong HS, Tang MHY, Yan KW, Cheung LWK (1999). Histological findings in a case of alobar holoprosencephaly diagnosed at 10 weeks of pregnancy. *Prenatal Diagnosis*.

[9] Wong HS, Lam YH, Tang MHY, Cheung LWK, Ng LKL, Yan KW (1999). First-trimester ultrasound diagnosis of holoprosencephaly: three case reports. *Ultrasound in Obstetrics and Gynecology*.

[10] Tongsong T, Chanprapaph P, Pongsatha S (1999). First-trimester diagnosis of conjoined twins: a report of three cases. *Ultrasound in Obstetrics and Gynecology*.

[11] Schwärzler P, Ville Y, Moscosco G, Tennstedt C, Bollmann R, Chaoui R (1999). Diagnosis of twin reversed arterial perfusion sequence in the first trimester by transvaginal color Doppler ultrasound. *Ultrasound in Obstetrics and Gynecology*.

[12] Econmides DL (1999). Early pregnancy screening for fetal abnormalities. *Ultrasound in Obstetrics and Gynecology*.

[13] Kanhai HHH, Keirse MJNC (1993). Low-dose sulprostone for pregnancy termination in cases of fetal abnormality. *Prenatal Diagnosis*.

[14] Hinshaw K, El-Refaey H, Rispin R, Templeton A (1995). Mid-trimester termination for fetal abnormality: advantages of a new regimen using mifepristone and misoprostol. *British Journal of Obstetrics and Gynaecology*.

[15] Dickinson JE, Evans SF (2003). A comparison of oral misoprostol with vaginal misoprostol administration in second-trimester pregnancy termination for fetal abnormality. *Obstetrics and Gynecology*.

[16] Dickinson JE (2005). Misoprostol for second-trimester pregnancy termination in women with a prior cesarean delivery. *Obstetrics and Gynecology*.

[17] Dickinson JE, Brownell P, McGinnis K, Nathan EA (2010). Mifepristone and second trimester pregnancy termination for fetal abnormality in Western Australia: worth the effort. *Australian and New Zealand Journal of Obstetrics and Gynaecology*.

[18] Dickinson JE, Doherty DA (2008). Factors influencing the duration of pregnancy termination with vaginal misoprostol for fetal abnormality. *Prenatal Diagnosis*.

[19] Nesbitt D, Giles W (1996). Prolonged induction to delivery time in termination of pregnancy using 16, 16-dimethyl-PGE1-methyl ester (gemeprost) for fetuses with a neural tube defect or hydrocephalus. *Australian and New Zealand Journal of Obstetrics and Gynaecology*.

[20] Lo TK, Lau WL, Lai FK (2008). Effect of fetal diagnosis on the outcomes of second-trimester pregnancy termination for fetal abnormalities: a pilot study. *Journal of Maternal-Fetal and Neonatal Medicine*.

[21] Wagner N, Abele H, Hoopmann M (2011). Factors influencing the duration of late first and second-trimester termination of pregnancy with prostaglandin derivates. *European Journal of Obstetrics Gynecology and Reproductive Biology*.

[22] Silva LV, Cecatti JG, Pinto E Silva JL, Amaral E, Barini R (2008). Feticide does not modify duration of labor induction in cases of medical termination of pregnancy. *Fetal Diagnosis and Therapy*.

[23] Lo TK, Lau WL, Lai FK (2008). Effect of fetal diagnosis on the outcomes of second-trimester pregnancy termination for fetal abnormalities: a pilot study. *Journal of Maternal-Fetal and Neonatal Medicine*.

[24] Say L, Kulier R, Gülmezoglu M, Campana A (2005). Medical versus surgical methods for first trimester termination of pregnancy. *Cochrane Database of Systematic Reviews*.

[25] Kulier R, Gülmezoglu AM, Hofmeyr GJ, Cheng LN, Campana A (2004). Medical methods for first trimester abortion. *Cochrane Database of Systematic Reviews*.

[26] Kapp N, Lohr PA, Ngo TD, Hayes JL (2010). Cervical preparation for first trimester surgical abortion. *Cochrane Database of Systematic Reviews*.

[27] Carbonne B, Brennand JE, Maria B, Cabrol D, Calder AA (1995). Effects of gemeprost and mifepristone on the mechanical properties of the cervix prior to first trimester termination of pregnancy. *British Journal of Obstetrics and Gynaecology*.

[28] Blanch G, Quenby S, Ballantyne ES, Gosden CM, Neilson JP, Holland K (1998). Embryonic abnormalities at medical termination of pregnancy with mifepristone and misoprostol during first trimester: observational study. *British Medical Journal*.

